# Supplementary material for: Enhanced responsivity and detectivity of fast WSe2 phototransistor using electrostatically tunable in-plane lateral p-n homojunction
Source: Nat Commun. 2021 Jun 7;12:3336. doi: 10.1038/s41467-021-23679-8 (PMC8185115; doi:10.1038/s41467-021-23679-8)
Supplement: Supplementary file 1 — Supplementary Information [file 41467_2021_23679_MOESM1_ESM.pdf]

Enhanced responsivity and detectivity of fast WSe_2 phototransistor using electrostatically tunable in-plane lateral p-n homojunction

Sayantan Ghosh[1], Abin Varghese[1,2,3], Kartikey Thakar[1], Sushovan Dhara[1], and Saurabh Lodha[1,*]

[1]Department of Electrical Engineering, IIT Bombay, Mumbai, 400076, India

[2]Department of Materials Science and Engineering, Monash University, Clayton, Victoria, 3800, Australia

[3]IITB-Monash Research Academy, IIT Bombay, Mumbai 400076, India

[*]email: slodha@ee.iitb.ac.in

Supplementary Figures 1-17

Supplementary notes 1-10

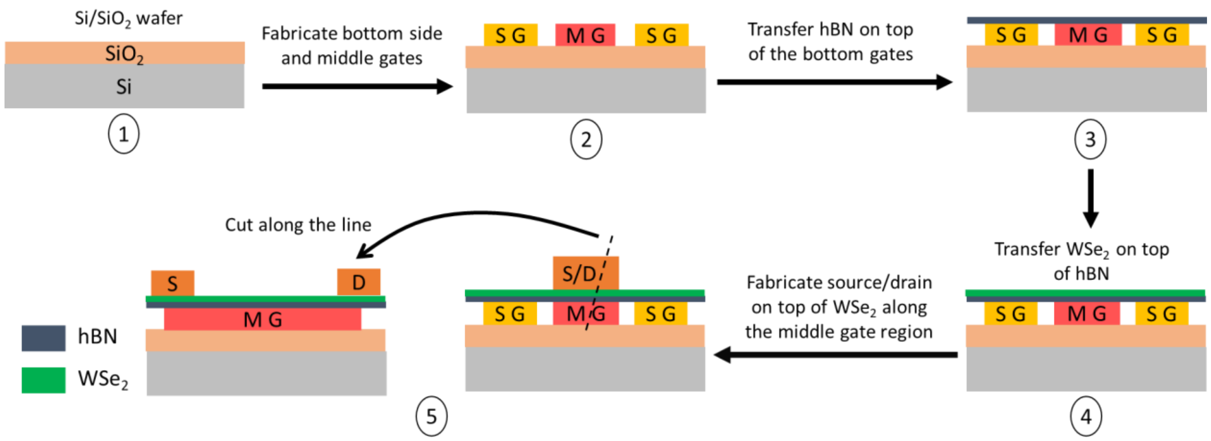

Supplementary Figure 1. Device fabrication steps are shown chronologically.

Supplementary note 1

In this note we have presented a simulation study of side gate architecture with an Si/SiO_2 system, as shown in Figure 2a. This device structure mimics the actual WSe_2/hBN device presented in this study. Si/SiO_2 was chosen since the material and physics models are well calibrated in TCAD software for this system. Although the dielectric constants for the materials are different, this mainly affects the quantitative extent of electrostatic doping keeping the qualitative trends the same, helping to realize the effect of side gates on phototransistor operation. In this structure we have used 20 nm thick SiO_2 as the gate dielectric and low doped p-type silicon of 10 nm thickness as the channel. Two metal side gates and a middle gate sandwiched between the two side gates were placed over the SiO_2. A metal substrate contact was added to the silicon channel to complete the electrical connections. Figure 2b shows the modulation of the silicon channel band diagram along the SG-MG-SG direction. Under fixed V_{MG} = -2 V, which keeps the MG channel region p-type, the side gate regions become more n-type as V_{SG} moves from 0 V to 1.5 V. This helps in efficient carrier separation with positive V_{SG}. Additionally, the hole density profile in Figure 2c shows a near 5% encroachment (100 nm from both sides for a 2 µm MG width) of the MG channel due to applied V_{SG} of 1.5 V. The encroachment may be higher in case of a WSe_2 channel that was used in our device. This is likely due to the lower dielectric constant of WSe_2 as compared to Si. Hence, the lateral p-n junction electric filed can penetrate further inside the middle gate channel of WSe_2 when compared to silicon, from the SG_MG direction. This reduction in MG channel width with positive V_{SG} reduces the dark current and enhances the detectivity as seen in the experimental data. Finally, Figure 2d shows the normalized photogenerated hole concentration (normalized against the photogenerated hole concentration at V_{SG} = 0 V) vs V_{SG} at a fixed V_{MG} = -2 V and for a fixed optical carrier generation rate that

resembles the laser power in our study. A monotonic increase in photogenerated hole concentration with increase in V_{SG} demonstrates the benefit of side gates in enhancing photocarrier population which increases the I_{ph} and responsivity of the device, as seen in the experimental data.

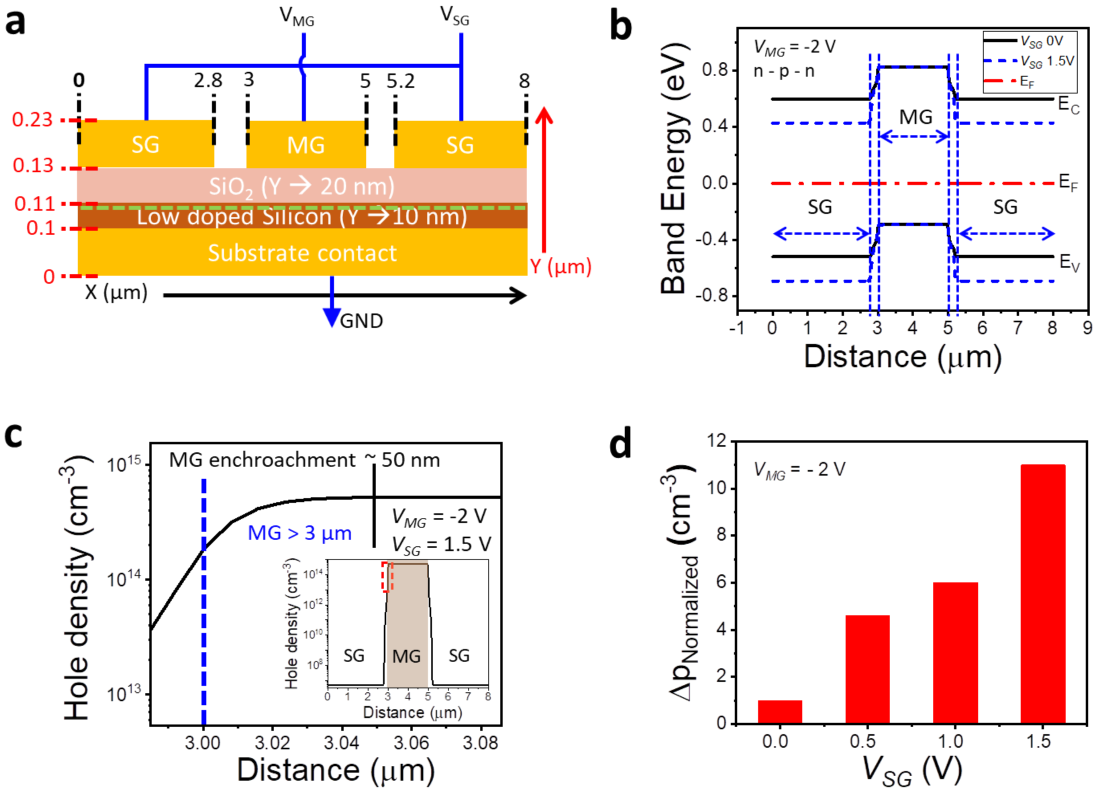

Supplementary Figure 2. TCAD simulations to illustrate device operation. a A representative schematic of the simulated device structure. **b** Band diagrams under fixed V_{MG} = -2 V and V_{SG} = 0 V and 1.5 V showing modulation of the energy bands to a stronger p-n configuration with increasing V_{SG}. **c** Hole density profile shows encroachment of MG channel by 50 nm from one side (100 nm from both sides) for a V_{SG} of 1.5 V. **d** A monotonic increase in photogenerated hole carrier density with increase in V_{SG} demonstrates the benefit of side gates.

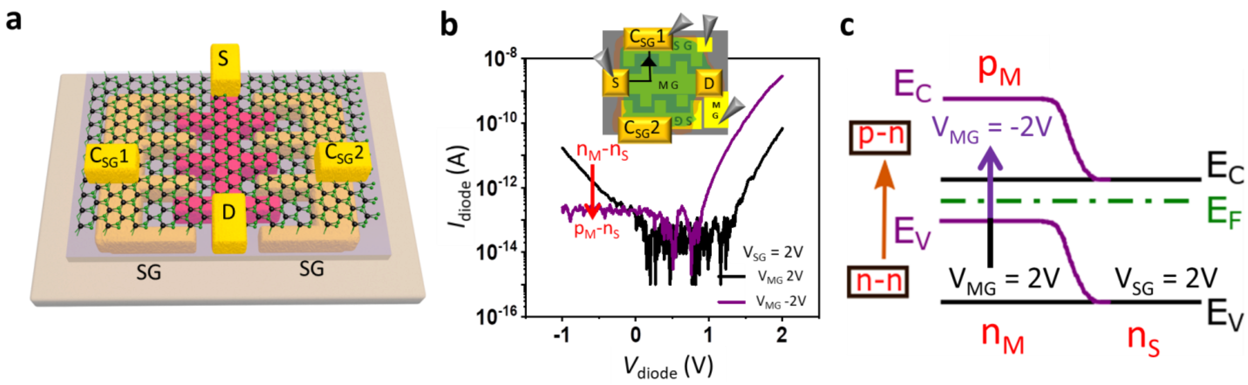

Supplementary Figure 3. a Schematic representation of WSe_2 FET with lateral p-n junction contacts on top (C_{SG1} and C_{SG2}). **b** Electrostatically doped, lateral (along the direction from MG to SG), in-plane p-n junction IV characteristics. IV curve changes from n_M-n_S to p_M-p_S configuration when V_{MG} is changed from 2 V to -2 V for a fixed V_{SG} = 2 V. **c** Energy band diagram schematic depicting localized doping concentration dependent change in configuration from n_M-n_S to p_M-p_S with change in V_{MG} from 2 V to -2 V for fixed V_{SG} = 2 V.

Supplementary note 2

The calculation of channel carrier density with varying gate voltage under No-V_{SG} condition (only MG is functioning) is presented below.

First, we have calculated hole mobility using the standard FET equation, $\mu = \frac{L}{W*V_D\left(\frac{\varepsilon_0\varepsilon_r}{d}\right)} \times \frac{dI_D}{dV_G}$, for linear region operation. Next, we have used the current equation, $I = p_{WSe2}*e*\mu*E*A$, to extract the values of p_{WSe2}, where p_{WSe2} is hole concentration in the channel, e is electronic charge in Coulomb, $E = \frac{V_D}{L}$, (V_D is drain voltage and L is the channel length between source and drain) is the electric field and A is the cross-sectional channel area. Here, we have used a constant μ ~ 5 cm^2/V-s obtained from the inversion region (beyond threshold voltage) over the entire range of V_{MG}.

Supplementary note 3

In this work, the MG FET channel area is less than the total device area. Enhanced photogeneration can be achieved if the total device area (area over SG-MG-SG) were to be considered as a single FET channel (Supplementary Figure 4). However, the three-gate (SG-MG-SG) structure is still superior due to the aspects discussed below. It is important to note that although we have reported both, shot noise-limited calculated *D** as well as flicker noise-limited measured *D** values in this work, for the sake of simplicity, we have assumed shot noise-limited *D** in this section.

(i) <u>Non-scalability of R and D* with Area:</u> It is possible to compare the responsivities between the only MG FET and the extended SG-MG-SG single FET structure. As per the Supplementary Figure 4 below, if we consider the MG channel length = L and its width = W, then the area above the middle gate (which takes part in S/D transport), $A = W*L$.

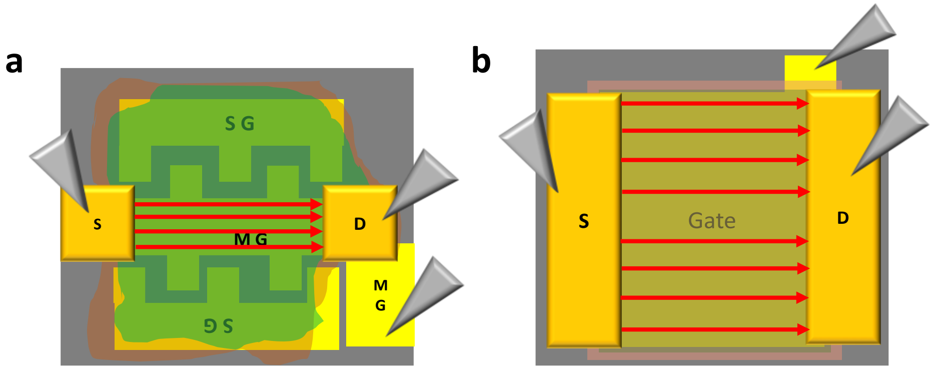

Supplementary Figure 4. Device architecture and carrier conduction path from source-to-drain are shown for a) the SG-MG-SG FET with only MG channel is active, and, b) the single extended gate FET structure.

Now, if we consider a larger area single FET with a width of 4W (as the total width including SG-MG-SG is approximately 4 times more than the MG width), then the area of the larger FET will be $A' = 4W*L = 4A$.

Considering extended S/D contacts as well, with the same voltage conditions (V_D and V_{MG}), the currents (dark and light) in the wider FET will be 4 times higher than the FET with only MG.

Now, as we know, responsivity, $R = I_{ph}/(P_{in}*(A_{device}/A_{laser}))$,

Then, for only MG device, $R1 = I_{ph}/(P_{in}*(A/A_{laser}))$, and,

for extended FET, $R2 = 4* I_{ph}/(P_{in}*(4*A/A_{laser})) = R1$. Hence, fundamentally we don't expect any difference in R between the extended FET and the FET only over MG.

Similarly, specific detectivity is expressed as, $D* = R\times \frac{A_{device}^{\frac{1}{2}}}{S_n}$

S_n from shot noise is expressed as, $S_n = 2qI_{dark}^{1/2}$

Then for only MG device operation, $S_{n1} = 2qI_{dark}^{1/2}$ and

for extended FET, $S_{n2} = 2q(4*I_{dark})^{1/2} = 2*S_{n1}$

Then, $D*_1 = R\times \frac{A^{\frac{1}{2}}}{S_{n1}}$ and,

for extended FET, $D*_2 = R\times \frac{2A}{S_{n2}} = D*_1$

(ii) <u>NPN (V_{SG} = 2 V) FET is still better than larger effective width (V_{MG}=V_{SG}) FET</u> Furthermore, we also present a comparison between No-V_{SG}, $V_{MG} = V_{SG}$ and V_{SG} = 2V conditions, and look at their effect on device responsivity and detectivity. In case of $V_{MG} = V_{SG}$, effectively the channel area expands, as the entire area above the MG and the SGs is under the same gate bias. Hence, in this case, a good number of carriers (dark or photo) participate in conduction and reach the S/D contacts. On the other hand, in case of No-V_{SG} condition, only the area above the MG takes part in S/D conduction, as shown in Supplementary Figure 5 below. Hence the $V_{MG} = V_{SG}$ case presents a close approximation to the single extended gate FET structure.

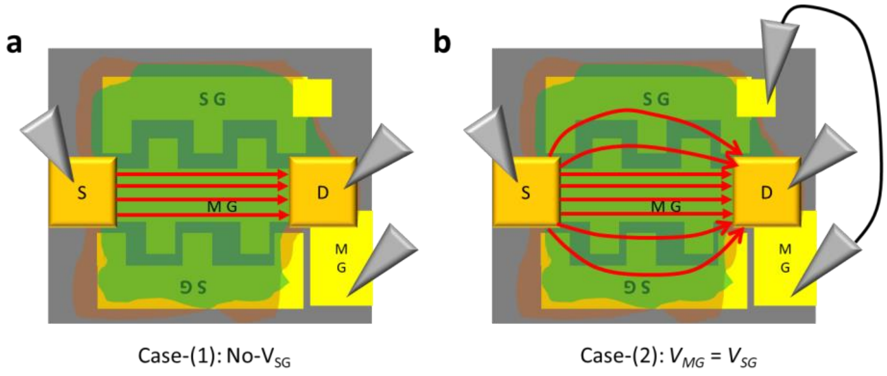

Supplementary Figure 5. Flow of holes from source-to-drain under transistor 'ON' state is shown for (a) No-V_{SG}, (b) $V_{MG} = V_{SG}$ [approximation to wider, single extended gate FET]

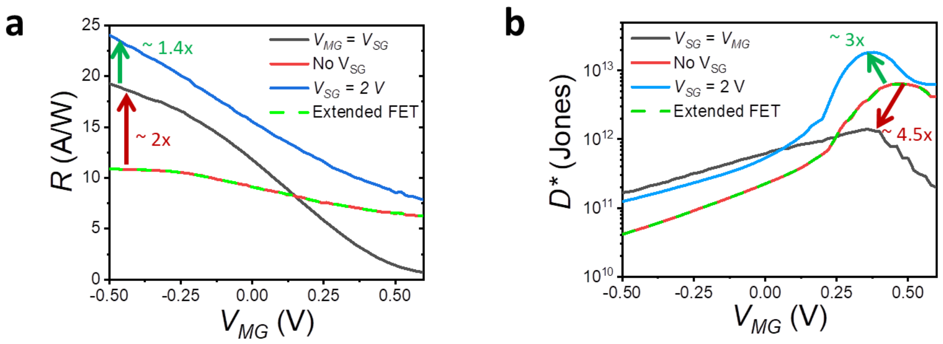

Supplementary Figure 6. Performance comparison between V_{SG} = V_{MG}, No-V_{SG} and V_{SG} = 2V conditions for (a) responsivity and (b) detectivity under P_{in} = 33 nW.

Although net responsivity for V_{MG} = V_{SG} condition is higher than the No-V_{SG} case as shown in Supplementary Figure 6, the maximum detectivity is much lower. Also, under applied V_{SG} = 2 V, in the three-gate configuration, both, responsivity and detectivity are higher than V_{MG} = V_{SG}. This shows the advantage of having separate SG electrical control instead of a single gate wide FET structure. Even with single SG we can expect an advantage in I_{ph} and R over no-SG architecture.

Advantage of single SG over no-SG: The two device SGs have been symmetrically fabricated with the same, continuous and uniform dielectric (hBN) and channel material (WSe_2) on top of the dielectric. Both the SGs are equidistant from the MG, fabricated simultaneously and operate independently of each other. Due to this, if a single SG is employed instead of two, it can be estimated that its effect will be reduced to half, with respect to the double SGs as shown in Supplementary Figure 7(a) schematic. A single SG would lead to a single p-n junction instead of two thereby reducing the carrier separation to half. With this consideration we have divided the responsivity and detectivity numbers by two and compared in Supplementary Figure 7(b).

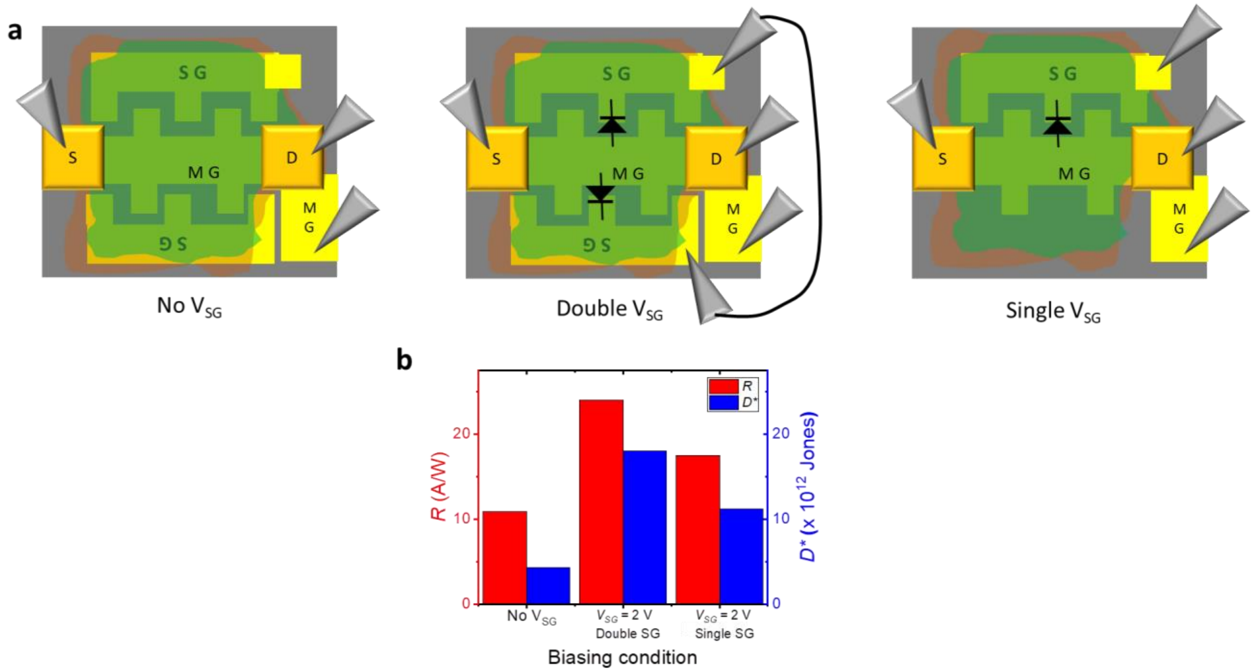

Supplementary Figure 7. The figure shows (a) electrical measurement connection configurations and formation of p-n homojunction for No-V_{SG}, double V_{SG} and single V_{SG} and (b) a comparison for maximum R and D^* for No-V_{SG}, and V_{SG} = 2 V with one and two side gates. Values for R and D^* presented in case of single side gate architecture are calculated based on the assumption discussed in the text. Supplementary Figure 7b shows that even with a single SG, the responsivity and detectivity numbers are still higher for positive V_{SG} than No-V_{SG}. This clearly shows the benefit of the lateral p-n homojunction in separating out electrons and holes and enhancing responsivity.

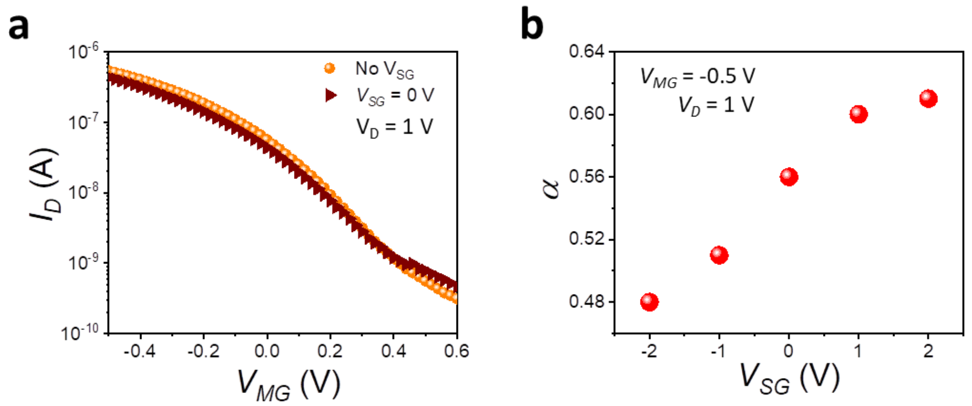

Supplementary Figure 8. a $I_D V_{MG}$ plots for 'No-V_{SG}' and 'V_{SG} = 0 V' are compared. Both IVs show very similar current through-out the V_{MG} range indicating that the side gates stay at nearly the same potential as V_{SG} = 0 V, when V_{SG} is not applied (No-V_{SG}). **b** Alpha increases monotonically with increasing (more positive) V_{SG}, indicating a decrease in the number of trap states participating in photoconduction.

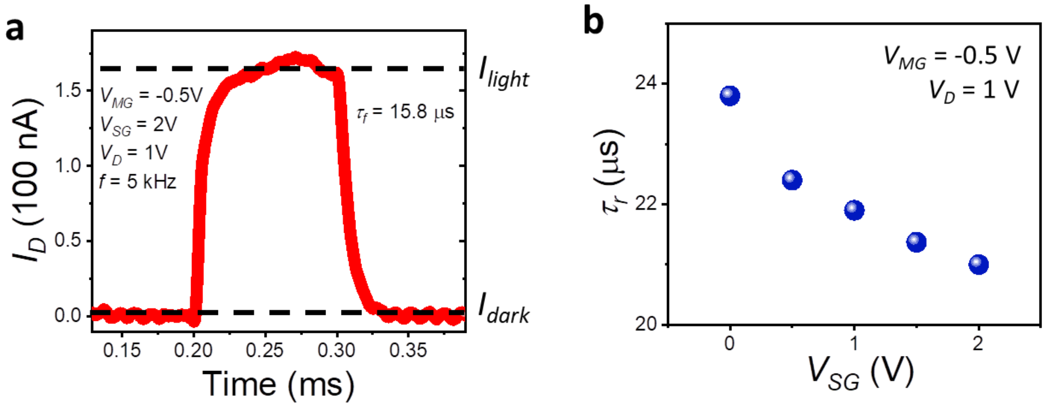

Supplementary Figure 9. a Transient photoresponse characteristics at V_{SG} = 2 V, V_{MG} = -0.5 V and an input light frequency of 5 kHz for an incident power of 33 nW. **b** Rise time vs V_{SG}. Rise time decreases monotonically with increasing V_{SG} due to increase in photogenerated hole concentration inside the S/D channel under n_S-p_M-n_S configuration, that helps in faster filling of trap states to reach steady state.

Supplementary note 4

Reasons for the difference in effective MG minority carrier (electron in this case) concentrations for case (1) and (3) are explained below.

Minority carrier concentration profile across a reverse biased MG-SG pn junction [case (2) in Fig. 4b of main manuscript] is shown in the Supplementary Figure 10. The channel over the MG region is the main region of interest since carrier conduction takes place primarily over the MG area. As it is well-known in pn junction theory that minority carriers get depleted in the quasi-neutral region near the boundary of the pn depletion region, the equilibrium (dark condition) electron carrier concentration in the MG region dips down near the depletion edge. When V_{MG} = -0.5 V is applied, and the channel becomes p-type, electrons act as minority carriers in the MG channel. The actual electron concentration profile (n_{p0} or $n_{2actual}$) is shown above. Now for simplification, if we consider a uniform and average electron concentration n_{2avg} throughout the MG channel then, $n_2 < n_1$, where n_1 is the electron concentration for case (1) in Fig. 4b of the main manuscript.

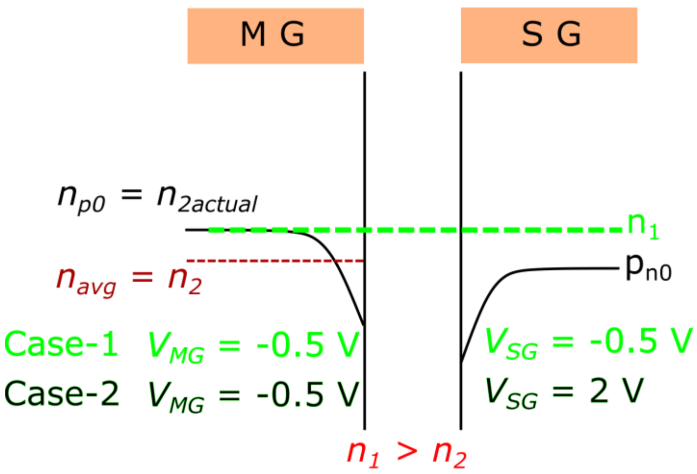

Supplementary Figure 10: Electron concentration profiles for two different V_{SG} conditions showing $n_1 > n_2$.

Supplementary note 5

Decrease in responsivity and fall time in a transistor from ON to OFF state: For V_{MG} = 0.4 V, the channel is n-type, mostly filled with electrons with holes acting as minority carriers, as shown in Figure 11. These electrons fill up most of the available long-lived electron trap states near the conduction band edge in dark condition. Hence, under illumination, very few photogenerated electrons will get a chance to occupy the low number of remaining unfilled electron trap states. Further, we know that, 1) under photogeneration same number of electron and holes are generated, and, 2) concentrations of photogenerated electrons and holes dominate over dark state concentrations. Thus, under illumination, there will be negligible imbalance in photogenerated electron and hole concentrations. Similar results have been reported for $MoSe_2$ phototransistors in reference 5. When the laser is turned off, the photogenerated electrons and holes recombine fast, leading to low τ_f (high speed). For V_{MG} = -0.5 V, the S/D channel has high hole concentration and low electron density (minority carriers). Hence most trap states near the conduction band are empty and available for photogenerated electrons to occupy. This leads to a photogating effect that increases τ_{life} for photogenerated holes resulting in larger G and R compared to V_{MG} = 0.4 V. When the laser is turned off, the trapped electrons slowly get de-trapped from the long-lived trap states to recombine with the photogenerated holes, which leads to large τ_f (slow speed).

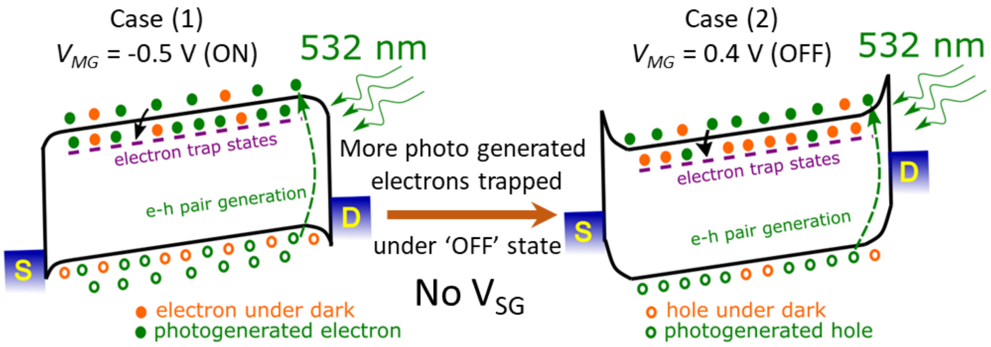

Supplementary Figure 11. Schematic band diagrams along S/D direction depicting electron, hole and available empty trap concentration for V_{MG} = -0.5 V (ON, heavily p-doped channel) and V_{MG}= 0.4 V (OFF, low n-doped channel) conditions. Larger empty trap concentration for V_{MG} = -0.5 V leads to larger lifetime, gain, R and τ_f compared to V_{MG} = 0.4 V.

Supplementary note 6

This section describes the methodology to calculate carrier lifetime (τ_{life}), transit time ($\tau_{transit}$), photoconductive gain (G) and external quantum efficiency (EQE).

τ_{life} values were extracted from exponential fits of the temporal decay characteristics, as shown in Figure 9 of this Supplementary Information. $\tau_{transit}$ is the time taken for a hole to travel from the source to the drain contact along the S/D channel. $\tau_{transit}$ was obtained using,

$$\tau_{transit} = \frac{L^2}{\mu V_D} \qquad \ldots\ldots\ldots\ldots\ldots\ldots\ldots\ (1)$$

where 'L' is the S/D channel length and μ is channel carrier mobility. Mobility was calculated from the $I_D V_G$ characteristics for No-V_{SG} and positive V_{SG} conditions (Fig. 2a, b) using,

$$\mu = \frac{L}{W \times V_D(\varepsilon_0 \varepsilon_r / d)} \times \frac{dI_D}{dV_G} \qquad \ldots\ldots\ldots\ldots\ldots\ldots\ldots\ (2)$$

Here W and d are the channel width and dielectric (hBN) thickness, respectively. ε_0 and ε_r are the dielectric constants for vacuum and hBN.

Fundamentally, hole mobility in the WSe_2 channel should not change with V_{SG}; however, the mobility values calculated from the on-currents show some variation with V_{SG} due to the varying channel width. As a result, $\tau_{transit}$ also varies with V_{SG}.

EQE is calculated using,

$$\text{EQE} = \frac{R}{G} \times \frac{hc}{\lambda q} \qquad \ldots\ldots\ldots\ldots\ldots(3)$$

where, h is Planck's constant, c is the speed of light, λ is the wavelength of incident light and q is electronic charge.

Supplementary note 7

Frequency dependent photocurrent (I_{ph}) measurements show a decrease in R and shot noise-limited calculated D^* (D^*_{shot}) with frequency in Supplementary Figure 12. This monotonic decrease in R and D^*_{shot} with frequency is consistent with literature.[1]-[2] This happens because of the presence of fast as well as slow electron traps in the MG channel region.

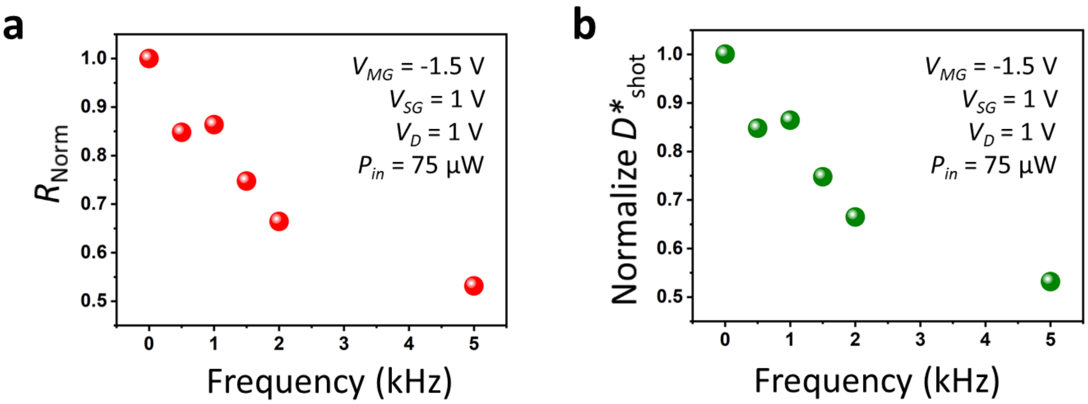

Supplementary Figure 12. Frequency dependent photoswitching characteristics. **a** Normalized R and **b** normalized shot noise-limited calculated D^* (D^*_{shot}) vs frequency. R and D^*_{shot} have been normalized to their DC values (at 0 Hz). Both R and D^*_{shot} show a monotonic decrease with frequency.

The slow electron traps near the conduction band edge may not be able to respond at 5 kHz frequency, hence there is reduced trapping of photogenerated electrons. Consequently, there is a reduction in photogating and gain with increasing frequency leading to lower photocurrent under AC (5 kHz) conditions and therefore a lower R ($R = \frac{I_{ph}}{P_{in}}$). A pictorial representation of electron and hole dynamics under DC and high frequency operation is presented in Supplementary Figure 13a and b.

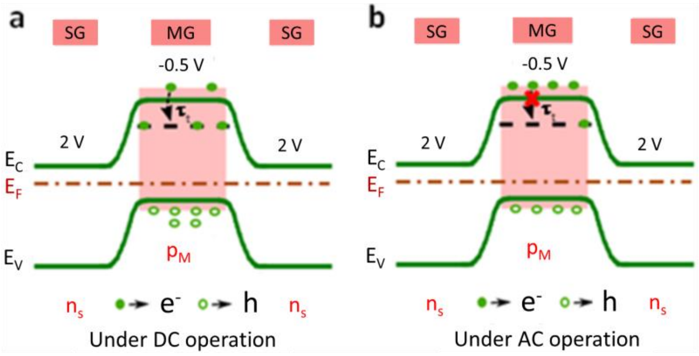

Supplementary Figure 13. Carrier dynamics for photogenerated electrons and holes are shown through band diagrams under (a) DC mode of operation (device is illuminated under a constant light source of 532 nm laser) and (b) under modulated light intensity switching from ON to OFF at high frequency (say, 5 kHz).

Supplementary note 8

This section highlights the differences in device performance under DC and AC operating conditions. Also, the advantages of the three-gate structure over a conventional single gate phototransistor under AC and DC operating conditions have been discussed.

***R* under AC operation can be lower than DC operation:** The value of R in Figure 4c and f is calculated from the photocurrent (I_{ph}) that is generated by an incident laser excitation at 5 kHz.

- *Prominent photogating and higher modulation in I_{ph} under DC operation:* In the main manuscript, we have discussed the impact of slow electron traps near the conduction band of WSe_2 and the effect of photogating. Under DC condition, electrons get trapped even in the slow electron trap states easily. Therefore, effectively, a greater number of photogenerated holes get accumulated and are collected by the contacts. This helps in achieving high I_{ph}.

- *Less photogating and poorer modulation of I_{ph} for AC operation:* In the case of AC operation at 5 kHz, photogating is not prominent as the trapping time is likely to be slower than 5 kHz. M. M. Furchi et al. and P. H. Hsu et al. have reported similar observations under high frequency operation [1], [2]. Now, such electron traps are present throughout the device in the WSe_2. That is, in WSe_2 over MG, over SG, as well as over the depletion region in-between MG and SG as shown in Figure 14. Therefore, lesser number of photogenerated holes will be available over SG and the area between SG and MG, to travel to the MG region (Figure 14b) under AC operation. Owing to this, the photocurrent does not reach its maximum value under 5 kHz photoswitching. The plot 'R_{Norm} vs frequency' also shows the same effect in Figure 12 of Supplementary Information. Also, because of this reason the modulation in R values with and without side gate at 5 kHz is lower than for DC operation.

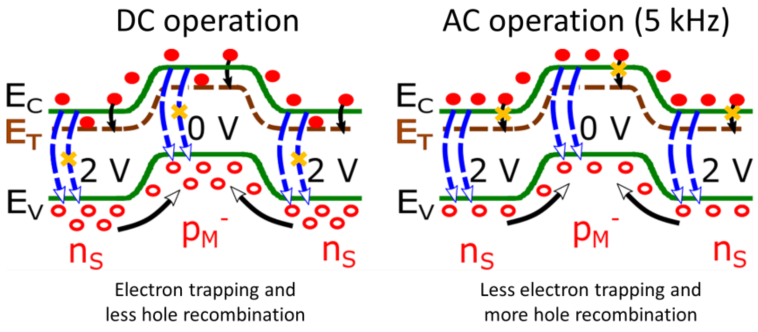

Supplementary Figure 14. WSe_2 band diagram along SG-MG-SG direction for DC and AC (5 kHz) operation. Under DC operation electron trap states get filled and the rate of e-h recombination decreases. This enables a large

number of holes to come in from SG to MG. High frequency (5 kHz) AC operation does not allow enough electron trapping resulting in a larger e-h recombination rate. This leads to a smaller number of holes travelling from SG to MG.

Advantage of the three-gate structure: Although the modulation in *R*, and its absolute value, for AC operation is smaller than for DC operation, there is a clear advantage for the three-gate structure under AC operation in the following cases.

- Advantages under AC operation:

1. *Less number of trap states in the channel material:* Less number of trap states can decrease the number of trapped photocarriers in the channel and thereby, weaken the photogating effect that causes the DC-AC imbalance. This will result in faster photo carrier dynamics [3] and extend the advantage of having three gates to a higher frequency.

2. *Modulating* V_{SG}*:* For a fixed trap density, one may achieve the same boost in *R* at high frequency (like DC) operation at a higher V_{SG}. Say, we get a 2x boost in R for V_{SG} = 1 V with respect to No-V_{SG}. Then, the same boost in R may be achieved in case of a fixed high frequency operation at V_{SG} = 2 V. Therefore, modulating V_{SG} can help in achieving the same *R* value at different operating frequencies which makes this device structure unique.

3. *Lower operating frequency can replicate DC operation:* For a fixed V_{SG} and trap density, a lower frequency of operation (in this study less than 5 kHz) in the three-gate device can give a boost close to DC operation. At lower frequencies, the effect of photogating increases.

Along with the aforementioned advantages this device architecture can provide few more advantages as listed below.

- Advantages under DC operation (as described in Supplementary note 3):

4. In this three-gate structure the device *R* can be modulated electrically with side gate voltage.

5. Lower off-current for three-gate structure vs single gate structure resulting in higher detectivity.

Supplementary note 9

Figure 15 shows the extraction method for noise equivalent power (NEP). NEP is the measure of minimum power required to obtain unity signal-to-noise ratio. In Figure 15 I_{ph} is plotted against P_{in} for V_{SG} = 2 V and at two different V_{MG} = ± 0.4 V (on/off condition of the transistor). The horizontal lines parallel to the X-axis represent measured noise power spectral densities (V_{MG} = -0.4 V (black line) and V_{MG} = 0.4 V (red line)) at 1 Hz frequency. The point of intersection for I_{ph} and the noise floor gives the NEP value. [4]

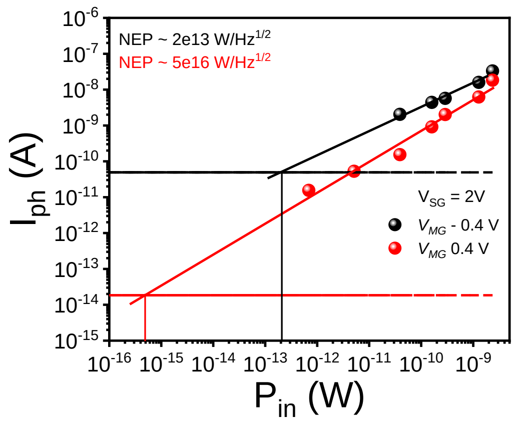

Supplementary Figure 15. Extraction of noise equivalent power (NEP) is shown for V_{SG} = 2 V for on/off conditions of the transistor corresponding to V_{MG} = ± 0.4 V. The NEP values are determined from the intersection points of the extrapolated fits of I_{ph} vs P_{in} with the measured noise floors under the same voltage conditions.

In figure 16, we have compared the measured D^* from flicker noise ($D^*_{flicker}$) with calculated D^* limited by shot noise (D^*_{shot}). This shows that, although the modulation in D^* from No-V_{SG} to V_{SG} = 2 V is very similar for both measured $D^*_{flicker}$ and calculated D^*_{shot}, D^*_{shot} overestimates the value of D^* by more than an order. Therefore, it can be said that flicker noise measurement is more appropriate to evaluate a phototransistor's D^*, instead of calculating from shot noise, especially in case of DC or very low frequency (typically <100 Hz) operation.

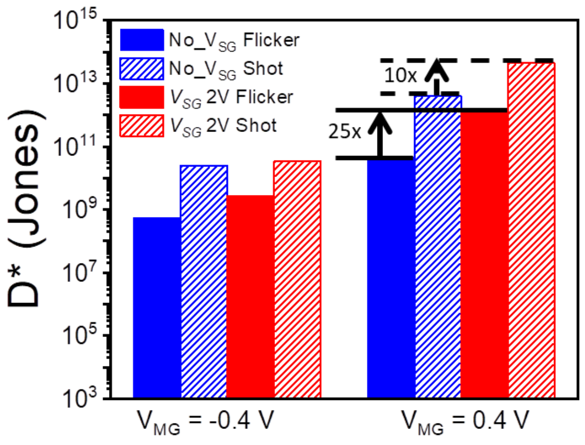

Supplementary Figure 16. A comparison of flicker noise- and shot noise-limited specific detectivity (D^*) under No-V_{SG} and V_{SG} = 2V, for V_{MG} = -0.4 V and 0.4 V

Supplementary note 10

The measurement of D* using flicker noise characterization was done on a separate but similar device (Device-2). Here a performance comparison of the two devices (Device-1 and Device-2) is presented.

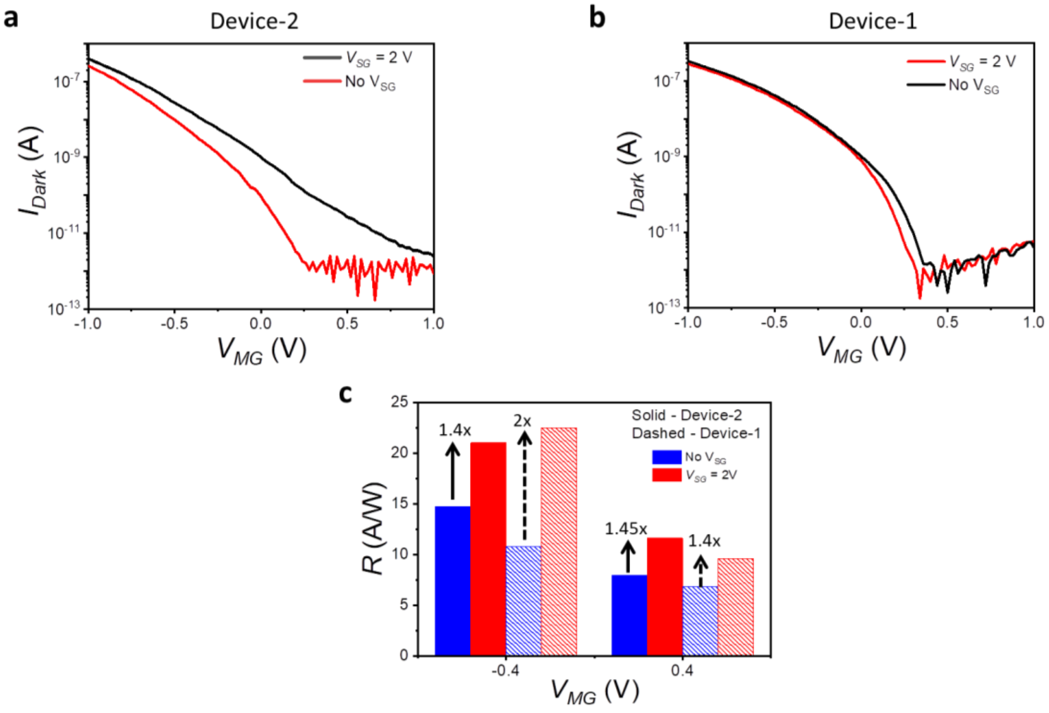

Supplementary Figure 17. I_DV_{MG} characteristics under dark for **a** Device-2 and **b** Device-1 for No-V_{SG} and V_{SG} = 2 V show a similar response in dark current to applied V_{SG}. **c** Photoresponsivity comparison under the same P_{in} = 33 nW at ±0.4 V (on/off condition) for No-V_{SG} and V_{SG} = 2 V confirms similar photoresponse under illumination.

Supplementary References:

1. Furchi, M. M., Polyushkin, D. K., Pospischil, A., & Mueller, T. Mechanisms of Photoconductivity in Atomically Thin $MoS0_2$. *ACS Nano. Lett.* vol. 14, pp. 6165 – 6170, (2014).
2. Hsu, H. P., Lin, D. Y., Jheng, J. J., Lin, P. C., & Ko, T. S. High Optical Response of Niobium-Doped WSe_2 layered crystal. *Materials.* vol. 12, no. 1161, pp. 1-8, (2019).
3. Chow, P. C. Y., Matsuhisa, N., Zalar, P., Koizumi, M., Yokota, T., & Someya, T. Dual-gate organic phototransistor with high-gain and linear photoresponse. *Nat. Commun.*, vol. 9, no. 4546, pp. 1–8 (2018).
4. Ravaro, M., Jagtap, V., Santarelli, G., Sirtori, C., Li, L. H., Khanna, S. P., Linfield, E. H., & S. Barbieri, Continuous-wave coherent imaging withterahertz quantum cascade lasers using electro-optic harmonic sampling, *Appl. Phys. Lett.*, vol. 102, no. 9, pp. 091107-1 - 091107-4, (2013).
5. Lee, H., Ahn, J., Im, S., Kim, J. & Choi, W. High-Responsivity Multilayer $MoSe_2$ Phototransistors with Fast Response Time. *Sci. Rep.*, vol. 8, pp. 11545, (2018).
